# Swarm and UNOISE outperform DADA2 and Deblur for denoising high-diversity marine seafloor samples

**DOI:** 10.1093/ismeco/ycae071

**Published:** 2024-05-09

**Authors:** Tonje Nilsen, Lars-Gustav Snipen, Inga Leena Angell, Nigel Brian Keeley, Sanna Majaneva, Ragnhild Pettersen, Knut Rudi

**Affiliations:** Faculty of Chemistry, Biotechnology and Food Science (KBM), Norwegian University of Life Sciences (NMBU), Chr. M. Falsensvei 18, Biotechnology Building, 1432 Ås, Norway; Faculty of Chemistry, Biotechnology and Food Science (KBM), Norwegian University of Life Sciences (NMBU), Chr. M. Falsensvei 18, Biotechnology Building, 1432 Ås, Norway; Faculty of Chemistry, Biotechnology and Food Science (KBM), Norwegian University of Life Sciences (NMBU), Chr. M. Falsensvei 18, Biotechnology Building, 1432 Ås, Norway; Institute of Marine Research, Dept. Tromso, P.O. Box 6606, Stakkevollan, 9296 Tromsø, Norway; Akvaplan-niva, Framsenteret, P.O. Box 6606, Stakkevollan, 9296 Tromsø, Norway; Akvaplan-niva, Framsenteret, P.O. Box 6606, Stakkevollan, 9296 Tromsø, Norway; Faculty of Chemistry, Biotechnology and Food Science (KBM), Norwegian University of Life Sciences (NMBU), Chr. M. Falsensvei 18, Biotechnology Building, 1432 Ås, Norway

**Keywords:** amplicon sequence variants, marine sediments, 16S rRNA gene

## Abstract

The performance of sequence variant resolution analytic tools for metabarcoding has not yet been adequately benchmarked for high-diversity environmental samples. We therefore evaluated the sequence variant tools DADA2, Deblur, Swarm, and UNOISE, using high-diversity seafloor samples, resulting in comparisons of 1800 sequence variant tables. The evaluation was based on 30 sediment grab samples, for which 3 replica samples were collected. Each replica sample was extracted using 5 common DNA extraction kits, resulting in 450 DNA extracts which were 16S rRNA gene sequenced (V3–V4), using Illumina. Assessments included variation across replica samples, extraction kits, and denoising methods, in addition to applying prior knowledge about alpha diversity correlations toward the cosmopolitan marine archaeon *Nitrosopumilus* with high diversity and the sulfide oxidizing *Sulfurovum* with low diversity*.* DADA2 displayed the highest variance between replicates (Manhattan distance 1.14), while Swarm showed the lowest variance (Manhattan distance 0.93). For the analysis based on prior biological knowledge, UNOISE displayed the highest alpha diversity (Simpson’s D) correlation toward *Nitrosopumilus* (Spearman rho = 0.85), while DADA2 showed the lowest (Spearman rho = 0.10). Deblur completely eliminated *Nitrosopumilus* from the dataset. For *Sulfurovum*, on the other hand, all the methods showed comparable results. In conclusion, our evaluations show that Swarm and UNOISE performed better than DADA2 and Deblur for high-diversity seafloor samples.

## Introduction

Metabarcoding has revolutionized the study of biodiversity in a range of fields [1]. Recently, the focus in metabarcoding studies has shifted toward obtaining sequence variant resolution, with DNA sequence error correction becoming a critical step [2]. Currently, the most widely applied tools for sequence error correction are DADA2 [3], Deblur [4], Swarm [5], and UNOISE [6]. The methods use different models and assumptions regarding sequencing errors or distribution of sequence variants, but they all arrive at some distinct sequence variants and assign a read count for these in each sequenced sample.

To validate the achievement of sequence variant resolution tools, benchmarking studies have been performed, e.g. [3, 7–9]. However, these benchmarking studies have not adequately included high-diversity samples, such as for prokaryotes at the seafloor, where potentially thousands of sequence variants may be present in a single sample [10, 11]. Furthermore, to our knowledge, Swarm has not yet been included in the benchmarking analyses.

Benchmarking on high-diversity samples, with unknown composition, remains a challenging task due to the lack of well-defined reference samples. However, benchmarking can still be performed using some very basic and general scientific principles. A fundamental principle in science is to apply replicates to derive technical variation [12]. Another principle involves using biological knowledge and reasoning about the systems investigated [13]. Prior knowledge could for instance be taxonomic entities associated with ecological patterns, such as alpha diversity.

In the case of the seafloor, we have recently shown a strong negative correlation between the genus *Sulfurovum* and alpha-diversity, while for the genus *Nitrosopumilus*, we observed a strong positive correlation with alpha-diversity for fish farming sites in Norway [11]. We linked these correlations to important biogeochemical processes at the seafloor, namely denitrification and nitrification, respectively. Similar correlations between denitrification and nitrification have also been made for waste-water treatment processes [14]. In the case of benchmarking, the link between alpha diversity and specific taxa/biogeochemical processes provides a valuable opportunity to assess DNA extraction and error correction methods from a biological perspective.

The aim of this paper was therefore to assess the sequence variant error correction tools for high diversity seafloor samples using (i) replica sampling including different DNA extraction approaches and (ii) biological reasoning based on prior knowledge about correlations between certain taxa and alpha diversity.

**Figure 1:**
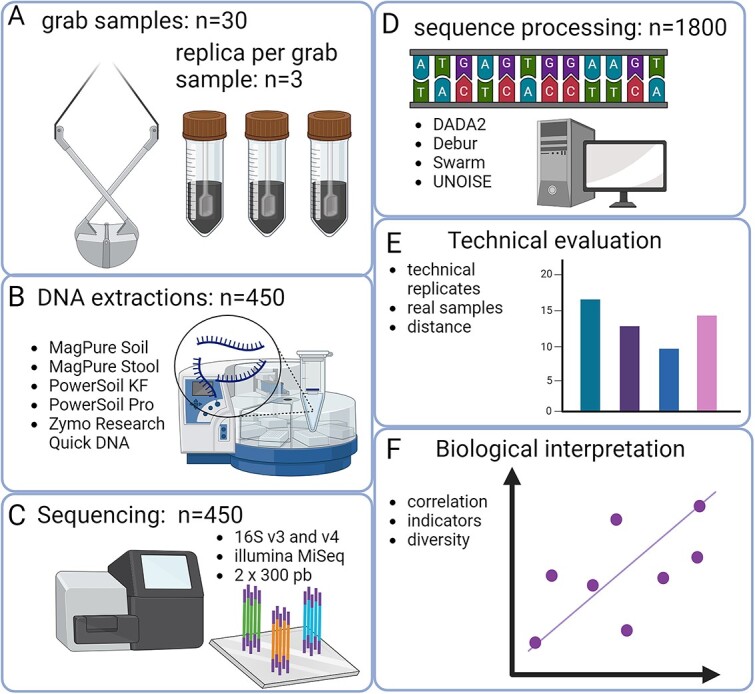
Experimental overview; (A) 30 grab samples were collected, with 3 technical replicates per grab, totalling 90 samples; (B) each of the 90 samples underwent DNA extraction using 5 distinct DNA extraction kits, resulting in 450 DNA extracts, and (C) the DNA extracts from each of the 450 samples were amplified with PCR primers targeting the V3 and V4 regions of the 16S rRNA gene; (D) subsequently, the 450 DNA sequence libraries were processed using 4 different processing strategies, leading to the creation of 1800 sequence variant tables; (E) technical variation was assessed based on the 15 replicates (3 extractions × 5 DNA extraction kits) per sample for the 4 sequence processing strategies; (F) correlations were performed to determine the relationships between alpha diversity and the genera *Sulfurovum* and *Nitrosopumilus*, with an anticipated negative correlation for *Sulfurovum* and a positive correlation for *Nitrosopumilus*.

Figure 1 presents an outline of the evaluation strategy employed in the current study.

## Materials and methods

From each of the 30 sediment samples, 3 parallels were subject to 5 different DNA extraction kits, and each sequencing data set was processed with 4 different 16S denoising pipelines or methods. This resulted in a total of 30 × 3 × 5 × 4 = 1800 read count profiles.

### Sediment samples

The samples in this study were part of a larger study of seafloor sediments around commercial fish farms along the Norwegian Coast (https://www.nmbu.no/forskning/prosjekter/aquaed). At each fish farm, samples were taken at various locations relative to the fish pens, and in most cases, two distinct “grabs” of sediments were taken at each such location. We refer to each such “grab” as a “sediment sample.” This data set has data from 30 such sediment samples (see Supplementary Table 1 for details). Each sediment sample was split into three parallels, and from each parallel, DNA was extracted using five different extraction kits: MagPure Soil DNA LQ kit, MagPure Stool DNA LQ kit, MagAttract PowerSoil DNA KF kit, MagAttract PowerSoil Pro DNA kit, and Zymo Research Quick-DNA Fecal/Soil Microbe 96 Magbead Kit. We will refer to these below as MagPure Soil, MagPure Stool, PowerSoil KF, PowerSoil Pro, and Zymo Quick-DNA.

### Sample preparation and DNA extraction

Samples were diluted 1:3 in STAR Buffer (Stool Transport and Recovery Buffer) and stored at −20°C until processing. All the extractions were done automatically by using a KingFisher Flex robot, due to extraction kits based on magnetic bead technology. The extractions mainly followed the manufacturers protocols.

The input varied slightly between the different kits, and 250-μL homogenized sediment sample were added to the PowerSoil Pro kit, PowerSoil KF kit, and MagPure Soil kit, and 200-μL sample were added to the Zymo Quick-DNA kit, and 150-μL sample were added to the MagPure Stool kit. All kits had a mechanical lysis step where we used a FastPrep 96. Samples extracted with PowerSoil Pro and PowerSoil KF were lysed in a bead plate with the following program: 4 × 30 s, 1800 rpm. Samples extracted with the three remaining kits were lysed in separate bead tubes, with the following program: 2 × 40 s, 1800 rpm. The rest of the extraction, for all five kits, followed the manufacturers protocols.

### PCR amplification and 16S rRNA library preparation

The preparation for 16S rRNA gene sequencing was identical for all the samples. Amplification of amplicons in the V3-V4 region was done using the PRK341 forward (5′-CCTACGGGRBGCASCAG-3′) and PRK806 reverse (5′-GGACTACYVGGGTATCTAAT-3′) primer [15], together with 1× HOT FIREPOL Blend Master Mix Ready to Load (Solis BioDyne, Estonia). The samples were amplified using the following program: 95°C for 15 min, 25 cycles of 95°C for 30 s, 55°C for 30 s, and 72°C for 45 s, followed by 72°C in 7 min. To purify the PCR products, we used 1× volume of Sera Mag Beads on the Biomek 3000 (Beckman Coulter, USA) and followed the manufacturers protocol. The samples were indexed with 1× FIREPol Master Mix Ready to Load (Solis BioDyne, Estonia) and a combination of 16 forward and 36 reverse primers, with Illumina indexes.

A normalized, pooled library was sequenced with Illumina MiSeq v3 at the Norwegian Sequencing Centre. The samples were sequenced on three different sequencing runs, where MagPure Stool and Zymo Quick-DNA were sequenced on a different run each, and PowerSoil Pro, PowerSoil KF, and MagPure Soil were sequenced together.

### Read processing and taxonomic assignment

We used four different software pipelines to process the data: DADA2 1.28.0 [3], Deblur 1.1.1 [4], Swarm 3.1.3 [5], and UNOISE [6], the latter as implemented in the VSEARCH software 2.22.1 [16]. Here, we describe the first processing of the reads, before the actual denoising.

First, reads were demultiplexed and barcodes removed. Then, the PCR primers (forward and reverse) were trimmed off the 5′ end of the read pairs (R1 and R2 reads). Next, raw reads were trimmed at their 3′ end. By trimming away the most error-prone part of each read, more reads survive the filtering/merging steps. A systematic test of degrees of trimming resulted in removing 20 bases off the R1 reads and 60 bases off the R2 reads as optimal for the current amplicons, see Supplementary Fig. 1 (see online supplementary material for a color version of this figure). This trimming was therefore used for all four processing methods.

Quality filtering and merging of read pairs differs between DADA2 and the other three methods. For Deblur, Swarm, and UNOISE, reads were preprocessed using VSEARCH. After trimming, read pairs were merged and quality filtered by using fastq_maxee_rate at 0.01. This implies that merged reads having an average error probability above .01 were discarded. In DADA2, merging is not done until after denoising. The quality trimming was done by setting maxEE to 2.75, which is 0.01 times the trimmed read length for R1 reads. We initially did the same for R2 reads, but this resulted in a majority of reads being discarded, and as suggested in the DADA2 manual (https://benjjneb.github.io/dada2/), we relaxed the R2 threshold to 0.02 times the read length (maxEE = 5.50).

The rest of the read processing was done with default settings for the four methods. This includes dereplication, denoising/clustering, and chimera detection. In the case of Deblur, all merged reads were trimmed to length 400. In the case of Swarm, only sequence variants where the centroid sequence had at least 2 copies were used, discarding the remaining sequence variants. This was done to employ some kind of abundance filtering in Swarm, since this is not done by default. All methods resulted in a fasta file with representative sequences for each sequence variant, and a table of read counts for each sequence variant in each sample.

All centroid sequences for all sequence variants, regardless of method used for finding them, were submitted to a taxonomic classification using the SINTAX algorithm as implemented in Vsearch 2.22.1, and its deployed RDP-based database [17]. Only sequences with confidence score above 0.8 at the genus rank were accepted as classified.

### Statistics

As mentioned above, a sediment sample (“grab”) was split into three replicates, and each of these were subject to five different DNA extraction kits. This produced 15 sets of reads from each of these 30 sediment samples. We expect the taxonomic composition in these 15 data sets to be very similar, being replicates of the same microbial community.

The four bioinformatic methods were used to process the reads from all these 15 × 30 data sets, and thus, each bioinformatic method produced 450 profiles of relative abundances. For each method, and for a given sediment sample, the 15 profiles were compared to each other to see how similar they were. We computed the Manhattan distance between all pairs, resulting in a 15 × 15 matrix of such distances. Then, the row average of this matrix was used as a measure of how much each profile differed from the other profiles within the same sediment sample. Again, since these 15 profiles originate from the same microbial community, the Manhattan distance between them should ideally be 0, and any variation introduced by either DNA extraction kits or bioinformatic method will be seen as increased Manhattan distances. This was then repeated for each of the 30 sediment samples and using profiles from each of the four bioinformatic methods.

We then used these Manhattan distances as the dependent variable in a linear mixed model, where the fixed effect of DNA extraction kit (5 levels) and bioinformatic method (4 levels) were estimated, using the sediment sample (30 levels) as a random effect. Thus, we have in this model 1800 response values (450 profiles obtained by the 4 different methods).

### Biological evaluation of alpha diversity

In the biological evaluations, we used prior knowledge from Norwegian fish farm sites indicating a negative correlation between the genus *Sulfurovum* and alpha diversity, as well as a positive correlation with the genus *Nitrosopumilus* [11].

To assess these relationships, we computed the sum of the relative abundances for sequence variants taxonomically assigned as *Sulfurovum* or *Nitrosopumilus*, respectively, for each of the 1800 read count profiles. We also computed the Simpson’s diversity index (D) for the exact same samples. Subsequently, we employed the nonparametric Spearman correlation method to correlate these summed relative abundances, with the logarithm (base 10) of Simpson’s diversity index (D) for each read count profile. For each read count profile, we also correlated the total read count number with the Simpson’s alpha diversity index.

## Results

### Library size

The influence of processing methods on library sizes is shown in Table 1. The results revealed a significant distinction among the extraction kits, with MagPure Stool yielding the highest number of reads and PowerSoil Pro producing the lowest. Regarding the processing methods, Deblur exhibited a significantly lower number of reads compared with the other approaches. While UNOISE generated the largest library sizes, the differences compared with Swarm and DADA2 were relatively minor.

**Table 1:** The average library size for each combination of DNA-kit/processing method.

**Extraction kit**	**Raw**	**DADA2**	**Deblur**	**Swarm**	**UNOISE**
MagPure Soil	66 463	44 720	12 551	52 743	59 483
MagPure Stool	99 948	67 153	19 895	74 870	86 823
PowerSoil KF	26 652	16 615	4976	21 182	24 120
PowerSoil Pro	10 740	6594	2311	8892	9850
Zymo Quick-DNA	21 984	11 135	4197	17 146	19 692

**Figure 2:**
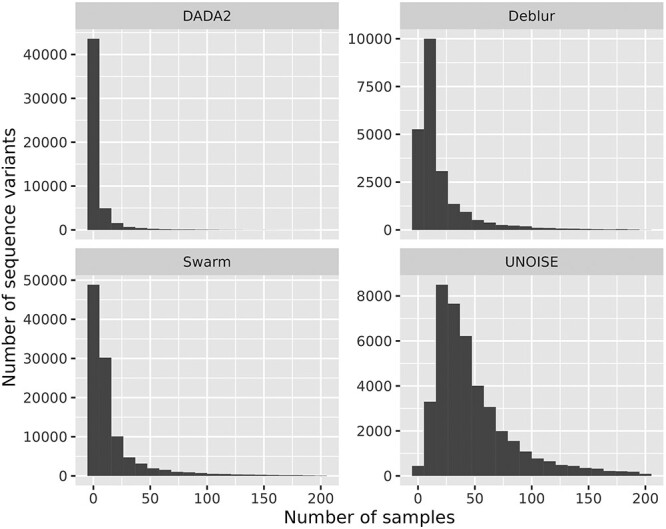
The histograms show the prevalence distribution of the sequence variants output by the various methods; the maximum possible prevalence (*x*-axis) is 450 since the data cover 450 samples.

The total number of sequence variants identified also varied substantially among the different processing methods evaluated. Deblur detected 22 903 sequence variants, UNOISE found 42 650, DADA2 found 52 369, and Swarm found 107 077 sequence variants from the exact same 450 samples of sequencing data. In Fig. 2, we display the prevalence of the sequence variants output by the various methods. In all cases, the majority of sequence variants are found in a small number of samples, which reflects the high diversity found in sediment samples. No method identifies sequence variants found in all 450 samples, but we observe that DADA2 has a maximum prevalence much lower than the other three methods.

### Variation across technical replicates

**Figure 3:**
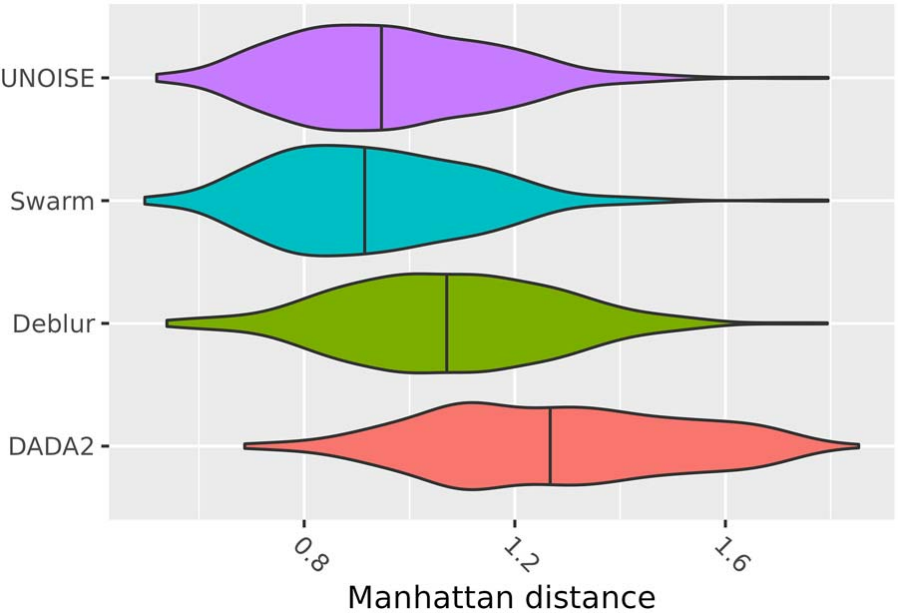
The observed Manhattan distances between profiles within each sediment-method group for the different methods; a larger Manhattan distance means larger variation between profiles within the same sediment sample, and the large variation for each method is 25%–55% due to the variation between the extraction kits and the sediment samples, and the line inside each violin plot marks the median value, and in the mixed model, all effects were estimated simultaneously, and the colors indicate all the four methods then came out significantly different (*P* < .01).

Although the true compositions of these samples are unknown, there are 15 sequencing data sets from each of the 30 sediment samples (3 parallels combined with the 5 DNA extraction kits). Regardless of the number of sequence variants identified by each method, ideally, the 15 profiles originating from the same sediment sample, and obtained by the same method, should exhibit considerable similarity. Since we only compared profiles of relative abundances, the variation quantified as Manhattan distances must range between 0.0 (identical profiles) and 2.0 (no overlap in sequence variants). In Fig. 3, we display boxplots showing the observed distances for the four bioinformatic methods. All of them are much larger than 0.0, indicating that the profiles are in general rather different within the same sediment samples. From the analysis-of-variance linear mixed model, we estimated the effects on these distances attributed to the various sources (extraction kits, methods, sediment samples). We found highly significant differences between all the four methods (*P* < .01) (Supplementary Table 2). The expected Manhattan distances were 0.93 for Swarm, 0.96 for UNOISE, 1.07 for Deblur, and 1.14 for DADA2. For the extraction kits, we found that PowerSoil Pro resulted in significantly smaller (0.95) and MagPure Soil a significantly larger (1.12) variation between replicates compared with the others, who came out similar (1.0–1.03).

### Alpha diversity relation to biological knowledge

**Figure 4:**
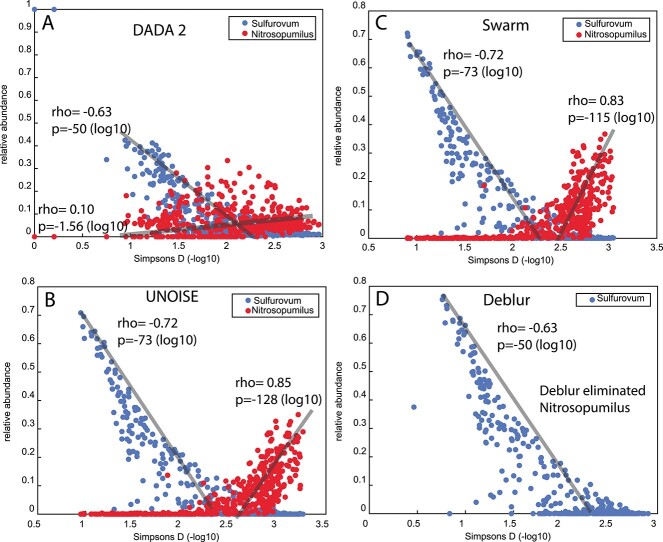
Associations between *Sulfurovum* and *Nitrosopumilus* with alpha diversity (Simpson’s D) was examined using four different methods: (A) DADA2, (B) UNOISE, (C) Swarm, and (D) Deblur; it should be noted that in the case of Deblur, no sequence variants belonging to *Nitrosopumilus* were identified, rendering a comparison impossible; the relative abundance for *Nitrosopumilus* is multiplied by 10, for visualization purposes.

In the biological evaluation as displayed in Fig. 4, we consistently observed a negative correlation between the relative abundance of *Sulfurovum* and alpha diversity measures as determined by 1-Simpsons D across all the sequence processing methods employed. This is in line with prior knowledge. However, the associations were slightly weaker for DADA2 and Deblur compared with Swarm and UNOISE. In contrast, we expected a positive correlation between diversity and the genus *Nitrosopumilus*. This was also clearly seen in the results from UNOISE and Swarm, but much weaker for DADA2, and for the Deblur, no sequence variants were recognized as being a representative of this genus at all. The results from Simpsons D were verified using the Shannon H alpha diversity measure. For *Sulfurovum*, the respective rho values for DADA2, Deblur, Swarm, and UNOISE were −0.71, −0.56, −0.66, and − 0.65, while for *Nitrosopumilus*, rho values for DADA2, Swarm, and UNOISE were −0.02, 0.84, and 0.84, respectively. Deblur failed to detect *Nitrosopumilus.*

For Simpsons D, all methods showed an expected positive correlation between diversity and distance to fish farm, with DADA2 showing the weakest correlation (Spearman rho = 0.40), followed by Deblur (Spearman rho = 0.43), Swarn (Spearman rho = 0.49), and UNOISE (Spearman rho = 0.51). Similar patterns were also obtained using Shannon H, with rho = 0.30 for DADA2, rho = 0.38 for Deblur, rho = 0.47 for Swarm, and rho = 0.47 for UNOISE.

### Taxonomic assignments

**Figure 5:**
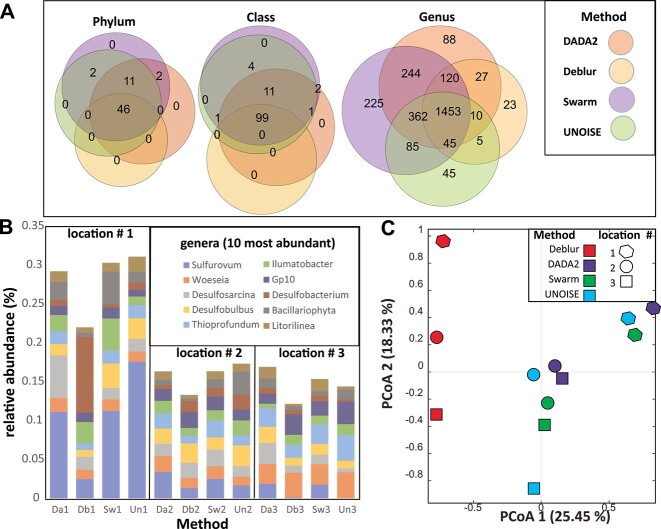
Taxonomic assignments; (A) Venn diagram showing the overlap in taxa between the processing methods at different taxonomic levels; (B) distribution of the 10 most abundant genera across location and processing method; (C) beta-diversity analyses based on Bray Curtis distances based on the genus distribution.

We finally evaluated if there were differences across the methods related to the taxonomic assignments. Deblur covered the lowest number of phyla, while Swarm covered the highest number (Fig. 5A). The same trend was observed for the Class and the Genus level, but less pronounced (Fig. 5A). The phyla not covered by Deblur are commonly newly discovered phyla, and phyla identified from extreme environments (Supplementary Table 3).

Both the taxonomic composition (Fig. 5B) and beta diversity analyses (Fig. 5C) at the genus level support that Deblur give deviating taxonomic assignments.

## Discussion

The main difference between our approach and previous benchmarking studies is that we focus on diversity rather than composition. We also focus on natural samples with unknown composition, but where diversity is known to be rather high. Thus, we cannot state anything about the methods ability to reconstruct the correct sequence variants, but since we have many data sets from the same microbial communities, we can investigate the reasons for an elevated variability between abundance profiles which ideally should be identical.

It is important to note that the effects detected for the extraction kits were confounded with sequencing runs, with the DNA from the kits MagPure Stool and Zymo Quick-DNA being sequenced on separate sequencing runs than for the other kits. Thus, we cannot conclude on the library sizes from the kits based on these results. The bioinformatic processing methods, on the other hand, are comparable since they are all working on all the data and thus has the exact same input. Here, we notice that Deblur filters away most reads, while the other three methods are quite similar in this respect. Note that the initial merging/filtering/dereplication were identical for Deblur, UNOISE, and Swarm, hence the differences between these three are from the denoising itself. Deblur requires that all merged reads are of the exact same length. We truncated all reads to 400 bases, which means that reads below this length is lost. However, this does not explain the reduced library sizes, since 93%–99% of the merged reads in the various samples were at least 400 bases before truncation. Instead, the loss of reads in Deblur is most likely due to the built-in requirement of reads being ”known 16S” sequences, i.e. a closed reference filtering. This makes the processing dependent on the supplied set of ”known 16S.” Sediment samples may contain many 16S variants currently not found in public databases. This requirement probably makes Deblur less suited for such data. This is also supported by the lack of taxonomic assignment of Deblur toward newly discovered phyla. Since the phyla not covered by Deblur are common in poorly characterized environments, this may also influence the conclusions from global microbial diversity surveys conducted within the frame of the Earth Microbiome Project, since Deblur was used for the sequence variant detection [18].

From Fig. 2, we can see that even the most prevalent sequence variants never reach much beyond half of the 450 samples. This is due to the high diversity in such environments. Still, the DADA2 has substantial lower prevalences than the other three methods. We might have expected that Deblur, having the lowest library sizes, would also have the lowest prevalences, but instead DADA2 stands out here. In Fig. 3, we display the data on the average Manhattan distances between profiles obtained by the various methods within the same sediment samples. The estimated values from the linear mixed model show the same overall picture but in addition show a significant difference between all methods. First, all values turned out quite large (0.93–1.14) indicating a large variation between profiles, which we believe is characteristic of high-diversity data in general. In high diversity data, all methods will come up with a large number of sequence variants, resulting in many (small) differences in the abundances that add up to a rather large Manhattan distance. In this perspective, it is perhaps surprising that Swarm, which identifies by far the most sequence variants in total, also has the smallest expected Manhattan distance. At the other end, DADA2 shows the largest variation between profiles. This is largely due to DADA2 listing many sequence variants unique to each single profile. The elevated variation observed for DADA2 across the replicates was surprising, as DADA2 represents the most computer intensive approach and was expected to perform better than the other methods in removing technical variation [3]. It is beyond the scope of this work to dig into the details of the algorithms behind these methods, but what clearly separates the DADA2 from the other three is how paired-end reads are merged. Both Deblur, UNOISE, and Swarm rely on merging the pairs early and then denoise. In DADA2, the forward and reverse reads are denoised separately and then finally merged without taking into account the pairwise information inherent in paired-end reads. In high diversity samples, this strategy may cause problems if the actual true sequences are very similar.

It should also be said that in many real-life analyses, very low abundant and/or low prevalent sequence variants would probably be discarded before any downstream analysis. This would reduce all differences we see between the methods here. In this context, one would then typically favor the fastest and simplest approach, which again are UNOISE and Swarm.

For the biological evaluation, we expected negative correlations between alpha diversity and *Sulfurovum* [11]*.* All methods consistently displayed this. For *Nitrosopumilus*, on the other hand, we expected a positive correlation [11]. However, only Swarm and UNOISE exhibited a strong positive correlation between *Nitrosopumilus* and alpha diversity. Deblur completely failed to detect *Nitrosopumilus*, while DADA2 showed only a rather weak positive correlation. The reason for Nitrosopumilus lacking from the Deblur sequence variants, we believe is most likely due to the built-in closed reference filtering.

The original intention of sequence variant analyses was to enable comparisons across samples and studies [2]. We believe that this idea relies on the somewhat unrealistic assumption that all types of errors can be effectively modeled. Our DNA extraction kit comparison suggests distinct error structures introduced by different extraction kits, and this is hardly accounted for in the denoising models. Coping with chemical modifications of DNA due to environmental conditions is even more challenging [19]. Furthermore, the resolution of sequence variants may not yield significant biological meaning, as most bacteria exhibit different sequence variants for their 16S rRNA gene [20]. We acknowledge that all tools used here have plenty of options for tuning their behavior and that a more extensive exploration of this may improve the results they produce. Most practitioners will, however, tend to stick to default settings as much as possible, which is why we started here. Further research in how to best deal with high-diversity metagenomes from outdoor environments should be encouraged.

In conclusion, our findings suggest that advanced sequence correction methods provided in DADA2 and Deblur may introduce more variation in high-diversity samples compared with simpler models such as Swarm and UNOISE, potentially removing biological patterns. Deblur also seems to eliminate environmentally important phyla. Therefore, it is essential for future bioinformatic pipeline development to carefully weigh the potential trade-offs associated with striving for greater sequence variant resolution through error correction. While the goal of achieving higher resolution may appear worthwhile, it is important to recognize that error correction algorithms may not fully capture the true error structure in data from natural environments. Consequently, it is crucial to maintain a focus on biological reasoning when designing bioinformatics pipelines, incorporating a thoughtful understanding of the limitations and assumptions associated with the chosen methods.

## Supplementary Material

Supplementary_figure_1_ycae071

Supplementary_Table_1_ycae071

Supplementary_Table_2_ycae071

Supplementary_Table_3_ycae071

## Data Availability

The raw sequencing data are available in the Sequence Read Archive (SRA) under the accession number PRJNA1034510.
