# Supplementary material for: Swarm and UNOISE outperform DADA2 and Deblur for denoising high-diversity marine seafloor samples
Source: ISME Commun. 2024 May 9;4(1):ycae071. doi: 10.1093/ismeco/ycae071 (PMC11170925; doi:10.1093/ismeco/ycae071)
Supplement: Supplementary_figure_1_ycae071 [file supplementary_figure_1_ycae071.docx]

**
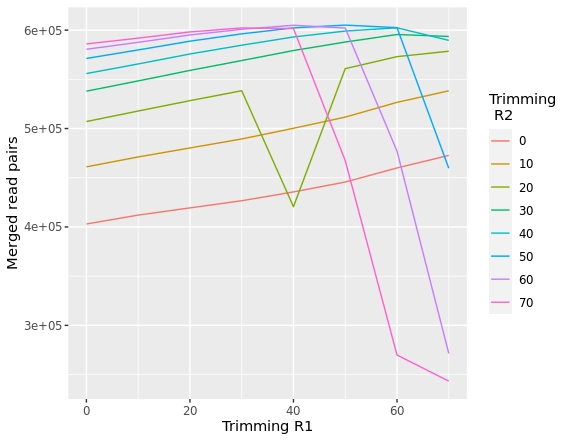
**

**Supplementary figure 1. The number of merged read pairs for a subset of samples as affected by trimming the 3’ end of R1 and R2 reads.** Trimming R1 of 20 bases in combination with trimming R2 by 60 gives many merged reads. Some even harder trimming give a few more merged reads, but experience tell us wrong merging emerges when overlap regions become too short
