# Supplementary material for: Swarm and UNOISE outperform DADA2 and Deblur for denoising high-diversity marine seafloor samples
Source: ISME Commun. 2024 May 9;4(1):ycae071. doi: 10.1093/ismeco/ycae071 (PMC11170925; doi:10.1093/ismeco/ycae071)
Supplement: Supplementary_Table_1_ycae071 [file supplementary_table_1_ycae071.docx]

**Supplementary Table 1. Information about grab samples**

| **Location #** | **Municipality** | **Sediment grab sample #** | **Depth (m)** | **Distance from fish farm (m)** |
| --- | --- | --- | --- | --- |
| Location 1 | Brønnøy | 1 | Unknown | Unknown |
|  |  | 2 | Unknown | Unknown |
|  |  | 3 | Unknown | Unknown |
| Location 2 | Gildeskål | 4 | 231 | 375 |
|  |  | 5 | 213 | 515 |
|  |  | 6 | 51 | 0 |
|  |  | 7 | 182 | 3760 |
|  |  | 8 | 231 | 375 |
|  |  | 9 | 203 | 275 |
|  |  | 10 |  |  |
|  |  | 11 | 51 | 0 |
|  |  | 12 | 182 | 3760 |
|  |  | 13 | 213 | 515 |
|  |  | 14 | 94 | 210 |
|  |  | 15 |  |  |
| Location 3 | Dønna | 16 | 52 | 30 |
|  |  | 17 | 84 | 25 |
|  |  | 18 | 31 | 441 |
|  |  | 19 | 27 | 225 |
|  |  | 20 | 84 | 94 |
|  |  | 21 | 51 | 64 |
|  |  | 22 |  |  |
|  |  | 23 | 30 | 535 |
|  |  | 24 | 41 | 200 |
|  |  | 25 | 50 | 912 |
|  |  | 26 | 31 | 441 |
|  |  | 27 | 84 | 25 |
|  |  | 28 | 31 | 335 |
|  |  | 29 | 50 | 912 |
|  |  | 30 | 52 | 30 |
