# Supplementary material for: Swarm and UNOISE outperform DADA2 and Deblur for denoising high-diversity marine seafloor samples
Source: ISME Commun. 2024 May 9;4(1):ycae071. doi: 10.1093/ismeco/ycae071 (PMC11170925; doi:10.1093/ismeco/ycae071)
Supplement: Supplementary_Table_2_ycae071 [file supplementary_table_2_ycae071.docx]

**Supplementary Table 2. Output from regression analysis.^1^**

^1^ The linear mixed model was fitted using the nlme R-package. A pairwise comparison (contrast) for the fixed effects was done using the multcomp R-package. The Estimate column are the estimates of the differences under Hypothesis, and the corresponding Tukey-corrected p-values are listed under Pr(>|z|).

| **Hypothesis** | **Estimate** | **Std. Error** | **z value** | **Pr(>\|z\|)** |
| --- | --- | --- | --- | --- |
| PowerSoil KF - Quick-DNA == 0 | -0.060741 | 0.009581 | -6.340 | <0.001 *** |
| PowerSoil Pro - Quick-DNA == 0 | 0.039122 | 0.009581 | 4.083 | <0.001 *** |
| MagPure Stool - Quick-DNA == 0 | -0.090014 | 0.009581 | -9.395 | <0.001 *** |
| MagPure Soil - Quick-DNA == 0 | -0.137993 | 0.009581 | -14.403 | <0.001 *** |
| PowerSoil Pro - PowerSoil KF == 0 | 0.099863 | 0.009581 | 10.423 | <0.001 *** |
| MagPure Stool - PowerSoil KF == 0 | -0.029272 | 0.009581 | -3.055 | 0.0189 * |
| MagPure Soil - PowerSoil KF == 0 | -0.077252 | 0.009581 | -8.063 | <0.001 *** |
| MagPure Stool - PowerSoil Pro == 0 | -0.129136 | 0.009581 | -13.478 | <0.001 *** |
| MagPure Soil - PowerSoil Pro == 0 | -0.177115 | 0.009581 | -18.486 | <0.001 *** |
| MagPure Soil - MagPure Stool == 0 | -0.047979 | 0.009581 | -5.008 | <0.001 *** |
| Deblur - Dada2 == 0 | -0.205641 | 0.008569 | -23.997 | <0.001 *** |
| Swarm - Dada2 == 0 | -0.348113 | 0.008569 | -40.623 | <0.001 *** |
| UNOISE - Dada2 == 0 | -0.316569 | 0.008569 | -36.942 | <0.001 *** |
| Swarm - Deblur == 0 | -0.142472 | 0.008569 | -16.626 | <0.001 *** |
| UNOISE - Deblur == 0 | -0.110928 | 0.008569 | -12.945 | <0.001 *** |
| UNOISE - Swarm == 0 | 0.031544 | 0.008569 | 3.681 | 0.00131 ** |
