# Supplementary material for: Swarm and UNOISE outperform DADA2 and Deblur for denoising high-diversity marine seafloor samples
Source: ISME Commun. 2024 May 9;4(1):ycae071. doi: 10.1093/ismeco/ycae071 (PMC11170925; doi:10.1093/ismeco/ycae071)
Supplement: Supplementary_Table_3_ycae071 [file supplementary_table_3_ycae071.docx]

**Supplementary Table 3. Phyla identified with the different processing methods.**

| **Phylum** | **DADA2^1^** | **Deblur^1^** | **Swarm^1^** | **Unoise^1^** |
| --- | --- | --- | --- | --- |
| Acidobacteria | 1 | 1 | 1 | 1 |
| Actinobacteria | 1 | 1 | 1 | 1 |
| Aminicenantes | 1 | 1 | 1 | 1 |
| Aquificae | 1 | 1 | 1 | 1 |
| Armatimonadetes | 1 | 1 | 1 | 1 |
| Atribacteria | 1 | 1 | 1 | 1 |
| BRC1 | 1 | 1 | 1 | 1 |
| Bacteroidetes | 1 | 1 | 1 | 1 |
| Balneolaeota | 1 | 1 | 1 | 1 |
| Campilobacterota | 1 | 1 | 1 | 1 |
| Candidatus_Saccharibacteria | 1 | 1 | 1 | 1 |
| Chlamydiae | 1 | 1 | 1 | 1 |
| Chloroflexi | 1 | 1 | 1 | 1 |
| Cloacimonetes | 1 | 1 | 1 | 1 |
| Cyanobacteria/Chloroplast | 1 | 1 | 1 | 1 |
| Deferribacteres | 1 | 1 | 1 | 1 |
| Deinococcus-Thermus | 1 | 1 | 1 | 1 |
| Dictyoglomi | 1 | 1 | 1 | 1 |
| Elusimicrobia | 1 | 1 | 1 | 1 |
| Fibrobacteres | 1 | 1 | 1 | 1 |
| Firmicutes | 1 | 1 | 1 | 1 |
| Fusobacteria | 1 | 1 | 1 | 1 |
| Gemmatimonadetes | 1 | 1 | 1 | 1 |
| Hydrogenedentes | 1 | 1 | 1 | 1 |
| Ignavibacteriae | 1 | 1 | 1 | 1 |
| Kiritimatiellaeota | 1 | 1 | 1 | 1 |
| Latescibacteria | 1 | 1 | 1 | 1 |
| Lentisphaerae | 1 | 1 | 1 | 1 |
| Marinimicrobia | 1 | 1 | 1 | 1 |
| Nitrospinae | 1 | 1 | 1 | 1 |
| Nitrospirae | 1 | 1 | 1 | 1 |
| Parcubacteria | 1 | 1 | 1 | 1 |
| Planctomycetes | 1 | 1 | 1 | 1 |
| Plantae | 1 | 1 | 1 | 1 |
| Poribacteria | 1 | 1 | 1 | 1 |
| Proteobacteria | 1 | 1 | 1 | 1 |
| Rhodothermaeota | 1 | 1 | 1 | 1 |
| SR1 | 1 | 1 | 1 | 1 |
| Spirochaetes | 1 | 1 | 1 | 1 |
| Synergistetes | 1 | 1 | 1 | 1 |
| Tenericutes | 1 | 1 | 1 | 1 |
| Thermodesulfobacteria | 1 | 1 | 1 | 1 |
| Verrucomicrobia | 1 | 1 | 1 | 1 |
| candidate_division_WPS-1 | 1 | 1 | 1 | 1 |
| candidate_division_ZB3 | 1 | 1 | 1 | 1 |
| Chlorobi | 1 | 0 | 1 | 1 |
| Chrysiogenetes | 1 | 0 | 1 | 1 |
| Crenarchaeota | 1 | 0 | 1 | 1 |
| Diapherotrites | 1 | 0 | 1 | 1 |
| Euryarchaeota | 1 | 0 | 1 | 1 |
| Microgenomates | 1 | 0 | 1 | 1 |
| Omnitrophica | 1 | 0 | 1 | 1 |
| Pacearchaeota | 1 | 0 | 1 | 1 |
| Thaumarchaeota | 1 | 0 | 1 | 1 |
| Thermotogae | 1 | 0 | 1 | 1 |
| Woesearchaeota | 1 | 0 | 1 | 1 |
| Abditibacteriota | 0 | 0 | 1 | 1 |
| Aenigmarchaeota | 1 | 0 | 1 | 0 |
| Caldiserica | 0 | 0 | 1 | 1 |
| Coprothermobacterota | 0 | 0 | 1 | 1 |
| candidate_division_WPS-2 | 1 | 0 | 1 | 0 |

^1^ Presence is indicated with 1, while absence is indicated with 0.
